# Species-rich ecosystems are vulnerable to cascading extinctions in an increasingly variable world

**DOI:** 10.1002/ece3.218

**Published:** 2012-04

**Authors:** Linda Kaneryd, Charlotte Borrvall, Sofia Berg, Alva Curtsdotter, Anna Eklöf, Céline Hauzy, Tomas Jonsson, Peter Münger, Malin Setzer, Torbjörn Säterberg, Bo Ebenman

**Affiliations:** 1 Division of Theoretical Biology, Department of Physics, Chemistry and Biology, Linköping UniversitySE-58183 Linköping, Sweden; 2 Ecological Modelling Group, Systems Biology Research Centre, Skövde UniversitySE-54128 Skövde, Sweden; 3 Department of Ecology & Evolution, University of ChicagoChicago, Illinois 60637; 4 Laboratoire Ecologie et Evolution, Université Pierre et Marie Curie75252 Paris Cedex 05, France

**Keywords:** Biodiversity, climate change, ecological networks, environmental variability, extinction cascades, food web, species interactions, stability, stochastic models, weather extremes

## Abstract

Global warming leads to increased intensity and frequency of weather extremes. Such increased environmental variability might in turn result in increased variation in the demographic rates of interacting species with potentially important consequences for the dynamics of food webs. Using a theoretical approach, we here explore the response of food webs to a highly variable environment. We investigate how species richness and correlation in the responses of species to environmental fluctuations affect the risk of extinction cascades. We find that the risk of extinction cascades increases with increasing species richness, especially when correlation among species is low. Initial extinctions of primary producer species unleash bottom-up extinction cascades, especially in webs with specialist consumers. In this sense, species-rich ecosystems are less robust to increasing levels of environmental variability than species-poor ones. Our study thus suggests that highly species-rich ecosystems such as coral reefs and tropical rainforests might be particularly vulnerable to increased climate variability.

## Introduction

The ecosystems of the world are increasingly exposed to and negatively affected by human-induced perturbations such as land degradation, overexploitation of natural resources, invasion of alien species, and climate change (Pereira et al. 2010). Theoretical as well as empirical work suggests that the response of ecosystems to such perturbations is governed by the pattern and types of interactions among species in the systems (May 1973a; Neutel et al. 2002; Ives and Cardinale 2004; O’Gorman and Emmerson 2009; Thebault and Fontaine 2010). Of particular current concern is the response of ecosystems to climate change (Petchey et al. 1999; Montoya and Raffaelli 2010; Maclean and Wilson 2011). Climate change involves both a change in mean conditions of climate variables and a change in their variability (Easterling et al. 2000). Climate data show and climate models predict that the frequency and intensity of weather extremes such as hurricanes (Bender et al. 2010), extreme precipitation events (Min et al. 2011), and heat waves (Meehl and Tebaldi 2004) have increased and will continue to do so if global warming increases as forecasted.

How will ecosystems respond to such increased levels of environmental variability caused by global warming? On the one hand, it has been suggested that environmental variation may, under certain conditions, facilitate the coexistence of competing species and hence promote species diversity (Chesson and Warner 1981; Gravel et al. 2011; see Adler et al. 2006; Shurin et al. 2010 for empirical work). Here, one necessary condition is that each species must be able to increase in abundance when rare—the so-called invasibility criterion (MacArthur 1972). One of the conditions for this criterion to be fulfilled is that species differ in their response to the environmental variability. On a similar note, it has been argued that intermediate intensity and frequency of disturbances might promote coexistence of competing species—the “inter-mediate disturbance hypothesis” (reviewed by Miller et al. 2011). High species diversity in turn should often have a stabilizing effect at the community level if species respond differently to environmental fluctuations, although stability at the species level might decrease (Tilman 1996; Yachi and Loreau 1999; Ives et al. 2000; Ives and Carpenter 2007; Gonzalez and Loreau 2009; Jiang and Pu 2009; Roscher et al. 2011). According to this view, biodiversity provides an insurance against system malfunction and collapse in a variable and unpredictable world (Yachi and Loreau 1999; Elmqvist et al. 2003). These findings are mainly based on theoretical analysis of simple model communities consisting of only one (or two) trophic level, assuming low levels of environmental variation and absence of demographic stochasticity and Allee effects, that is, factors that make small populations vulnerable to extinction.

On the other hand, increased environmental variability can be expected to result in increased variation in the fecundity and survival rates of species causing population stability and long-run growth rates of populations to decrease (Boyce et al. 2006; Morris et al. 2008). In combination with demographic stochasticity and Allee effects, this might lead to increased extinction risks of populations and species in ecosystems (May 1973a; Ruokolainen et al. 2007; Adler and Drake 2008; Borrvall and Ebennman 2008; Ruokolainen and Fowler 2008; Gravel et al. 2011; for experimental work see Shurin et al. 2010; Violle et al. 2010; Burgmer and Hillebrand 2011). The loss of one species might in turn trigger a cascade of secondary extinctions (e.g., Pimm 1980; Borrvall et al. 2000; Dunne et al. 2002; Ebenman et al. 2004; Eklöf and Ebenman 2006; Petchey et al. 2008; Dunn et al. 2009; Dunne and Williams 2009; Fowler 2010; Stouffer and Bascompte 2011), the extent and risk of extinction cascades being dependent on the structure of the community, such as its species richness and connectance, and on the characteristics of the species initially lost (reviewed by Ebenman and Jonsson 2005; Montoya et al. 2006; Ebenman 2011).

Thus, theoretical work suggests that increased levels of environmental variability might either facilitate or impede the long-term coexistence of interacting species. Results from empirical studies are conflicting; for instance, temperature variability has been found to promote greater species richness in zooplankton communities in lakes (Shurin et al. 2010) while reducing species richness and increasing extinction rates in microcosm phytoplankton communities (Burgmer and Hillebrand 2011). How ecosystems will respond to increased levels of environmental variability caused by global warming and how this response will be mediated by biodiversity is therefore, to a large extent, an open question. Here, we address this pressing question by investigating the dynamics of multitrophic model ecological communities exposed to high levels of environmental variation. Specifically, we explore how species richness and degree of correlation among species in their responses to environmental fluctuations affect the risk and nature of extinction cascades. We hypothesize that extinction cascades will occur more frequently in species-rich food webs than in species-poor ones. This is because mean densities of species tend to be lower in species-rich ecosystems than in species-poor ones due to increased intensity of competition—density compensation (reviewed by Gonzalez and Loreau 2009)—and lower densities should in turn lead to higher extinction risks. We also investigate how species richness and degree of correlation among species in their response to environmental fluctuations affect the temporal stability of total (aggregate) abundance of primary producers. We examine two scenarios: one where consumer species are generalists and one where they are specialists. Our approach is theoretical: we generate topologically feasible model food webs that are persistent in a deterministic, constant environment. The response of these food webs to high levels of environmental variation is then analyzed using generalized Rosenzweig–MacArthur models (Rosenzweig and MacArthur 1963) with stochastic parameters and saturating (type II) functional response of consumers. Demographic stochasticity and potential Allee effects are accounted for by introducing quasi-extinction thresholds.

## Material and Methods

We consider triangular food webs (i.e., decreasing number of species with increasing trophic level) with three trophic levels: primary producers, herbivores (primary consumers), and carnivores (secondary consumers). We vary the number of species (*s*) in the webs from six to 24 species while keeping the proportion of species at the different trophic levels the same in webs of different sizes. Connectance (*C*)—here defined as the number of trophic (consumer-resource) links (*L*) divided by the number of species raised to 2 (*s*^2^) (i.e., *C*=*L*/*s*^2^)—is kept constant at a value of 0.14, which is within the range (∼0.03–0.3) observed for real food webs (e.g., Dunne et al. 2002). A constant connectance means that the average number of links per species (link density) increases with increasing species richness. Trophic links are randomly allocated between species at different trophic levels subject to the following constraints: herbivores must feed on at least one basal species; carnivores must feed on at least one herbivore. Carnivores are potentially omnivorous. Consumers are either specialists (strong preference for one resource species) or generalists (equal preference for each of their resource species). There are also nontrophic interactions present: each primary producer species directly compete with all other primary producer species and direct intraspecific competition is present in all species.

Food web dynamics are described by a generalized Rosenzweig–MacArthur model with stochastic parameters (Rosenzweig and MacArthur 1963; Borrvall and Ebenman 2008): 

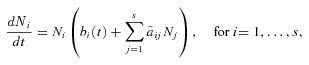

where 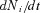 is the rate of change of density of species *i* with respect to time in a community with *s* species, *b_i_*(*t*) is the intrinsic per capita growth (mortality) rate of primary producer (consumer) species *i* at time *t*, and 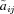 is the per capita effect of species *j* on the per capita growth rate of species *i*. The functional response of consumers is of type II, meaning that the per capita strength of trophic links, 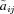, is a function of the densities of resource species (see Appendix for details). Preliminary analyses with type I and type III functional responses show that our results are robust with respect to the type of functional response (type I, II, and III). We introduce environmental stochasticity as white noise (i.e., no serial correlation) in the intrinsic growth rates; 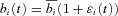, where 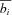 is the mean value of the intrinsic growth rate of species *i* and 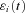 is a stochastic variable drawn from a uniform distribution with minimum, mean, and maximum values equal to −1, 0, and 1, respectively. This gives a variance in ɛ of 0.333 with extreme values as likely as the mean value. We use such a distribution because the aim of our study is to explore how communities respond to highly variable environments in which extreme values are likely to occur. The correlation, ρ, among species in their response to environmental fluctuations is varied from ρ= 0.1 to ρ= 0.9 in steps of 0.2 (see Appendix for a detailed description of the model).

To attain some generality, we generate a large number of replicate communities with constrained randomization of links and parameters (see Appendix for details of parameterization). We keep generating replicates until 200 communities that are persistent in a deterministic environment have been found. Then, each of the 200 replicate communities is exposed to environmental stochasticity for a period of 10,000 time units. A species is considered extinct if its density falls below a specified quasi-extinction threshold. Defining quasi-extinction thresholds is a way of accounting for processes such as demographic stochasticity, inbreeding depression, and potential Allee effects. The strength of demo-graphic stochasticity has been found to decrease with decreasing intrinsic growth/mortality rates and increasing generation time of species (Sæther et al. 2004; see also Pimm 1991, ch. 7). In our webs (as well as in many real webs; see Appendix), species’ intrinsic growth rates decreases and generation time increases with increasing trophic level. The quasi-extinction threshold was therefore set higher for basal species than for top predators (2 × 10^–3^ for basal species, 10^–4^ for herbivores, and 10^–5^ for carnivores).

The time of each extinction event is recorded and the probability of extinction for species at different trophic levels calculated. We also calculate the temporal stability of the aggregate abundance of all primary producers. As a measure of temporal stability, we use the reciprocal of the coefficient of variation (i.e., 1/CV) of the aggregate abundance of all primary producers over time. We also measure the degree of synchrony in the per capita growth rates of species (see Appendix).

## Results

**Figure 1:**
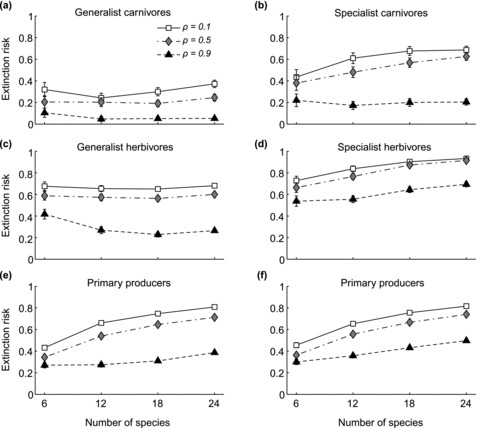
Mean per species risk of extinction (bars show 95% CI) for carnivores (a, b), herbivores (c, d), and primary producers (e, f) as a function of number of species in the food web. Left column (a, c, e) shows results for food webs with generalist consumers and right column (b, d, f) shows results for webs with specialist consumers. Series display the degree of correlation in species responses to environmental variation, ρ; ρ= 0.1 (solid line), ρ= 0.5 (dash-dotted line), and ρ= 0.9 (dashed line). Scenario: high environmental variation (var(ɛ) = 0.33) and weak intraspecific competition in consumers (*a_ii_=*–0.001). Results based on 200 independent replicate model food webs. Bars show 95% CI.

**Figure 2:**
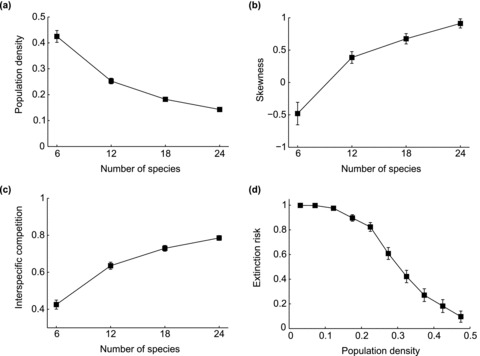
(a) Mean population density and (b) skewness of species density distribution of primary producers as functions of species richness, (c) intensity of interspecific competition as a function of species richness, and (d) extinction risk of primary producers as a function of their initial density. In (d), densities* of primary producers are divided into 10 classes (from 0 to 0.5 in steps of 0.05). For each class, the mean population density and mean risk of extinction following a stochastic simulation are given. Scenario: low correlation in species responses to environmental variation (ρ= 0.1), high environmental variation (var(ɛ) = 0.33), and weak intraspecific competition in consumers (*a_ii_=*–0.001). Results based on 200 independent replicate model food webs. Bars show 95% CI. Notes: Densities are the final densities resulting from simulation over 50,000 time units in a deterministic setting (constant environment) using the mean intrinsic growth rates (mortality rates) of species. These densities are then used as initial (starting) densities in the stochastic simulation. Intensity of interspecific competition experienced by producer species *i* is measured as sum(*ã_ij_N_j_*), where *j* are primary producer species competing with *i*.

**Figure 3:**
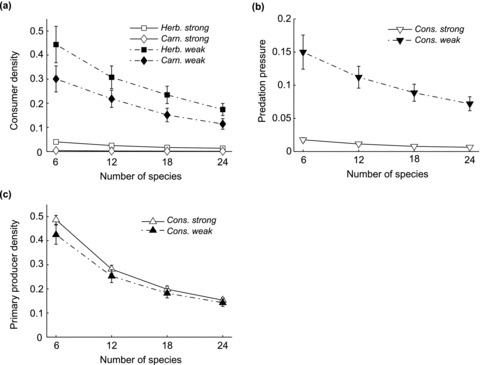
(a) Mean population density of herbivores and carnivores, (b) predation pressure on primary producers, and (c) mean population density of primary producers as functions of species richness and strength of intraspecific competition in herbivores and carnivores. Scenario: low correlation in species responses to environmental variation (ρ= 0.1) and high environmental variation (var(ɛ) = 0.33). Results based on 200 independent replicate model food webs. Filled symbols reperesent weak intraspecific competition and open symbols represent strong intraspecific competition. Bars show 95% CI. Notes: Densities are the final densities resulting from simulation over 50,000 time units in a deterministic setting (constant environment) using the mean intrinsic growth rates (mortality rates) of species. These densities are then used as initial (starting) densities in the stochastic simulation. Predation pressure experienced by producer species *i* is measured as sum(*ã_ij_N_j_*), where *j* are consumer species feeding on *i*.

**Figure A1:**
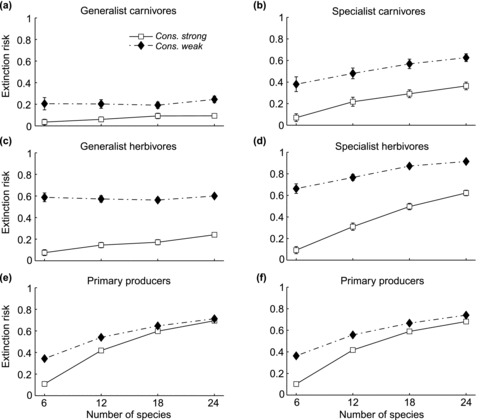
Mean per species risk of extinction (bars show 95% CI) for carnivores (a, b), herbivores (c, d), and primary producers (e, f) as a function of the number of species in the web. Left column (a, c, e) shows results for webs with generalist consumers and right column. (b, d, f) shows results for webs with specialist consumers. Series display the strength of intraspecific competition in consumers; strong (solid line) and weak (dash-dotted line). Scenario: high environmental variation (var(ɛ) = 0.33) and intermediate correlation among species in their response to environmental fluctuations (ρ= 0.5). Results based on 200 independent replicate model food webs.

The extinction risk of primary producers increases with increasing species richness (Fig. 1). Moreover, the mean population density of primary producers decreases with increasing species richness (Fig. 2a). Also, the proportion of rare species increases with increasing species richness. That is, the species-abundance distribution is more skewed to the right in species-rich food webs compared to species-poor ones (Fig. 2b). A partial explanation for the low densities in species-rich communities is the high intensity of interspecific competition in species-rich communities (Fig. 2c). Thus, primary producers will be closer to the extinction threshold in species-rich than in species-poor food webs resulting in increased risk of extinction (Fig. 2d). Furthermore, for a given species richness, primary producers are closer to the extinction threshold when the strength of intraspecific competition in consumer species is weak compared to when it is strong (Fig. 3). Weak intraspecific competition in consumer species therefore leads to higher extinction risks of primary producers compared to when intraspecific competition is strong (see Fig. A1; this is also supported by results from regression tree analysis, Table A1).

**Table A1:** Regression tree explaining the variation in extinction risk of primary producers. Predictors: correlation among species in their response to environmental variation; prey preferences of consumers; species number; and strength of intraspecific competition in consumers. Overall, the regression tree explains 51.7% of the variation in the extinction risk.

Split								Variable and value(s)	Number of observations	Extinction risk
0								All data	24,000	0.464
	1							Species number < 9	1,6000	0.221
		2						Intraspecific competition < –0.05	1,4800	0.190
		2						Intraspecific competition ≥–0.05	1,1200	0.346
	1							Species number ≥ 9	18,000	0.544
			3					Species number < 15	1,6000	0.435
				4				Correlation ≥ 0.8	1,1000	0.298
						6		Preference ≥ 0.5 Generalists	21,600	0.150
						6		Preference < 0.5 Specialists	11,400	0.520
				4				Correlation < 0.8	1,5000	0.462
			3					Species number ≥ 15	12,000	0.599
					5			Correlation ≥ 0.8	1,2000	0.442
							7	Preference ≥ 0.5 Generalists	1,1200	0.289
							7	Preference < 0.5 Specialists	11,800	0.670
					5			Correlation < 0.8	10,000	0.631
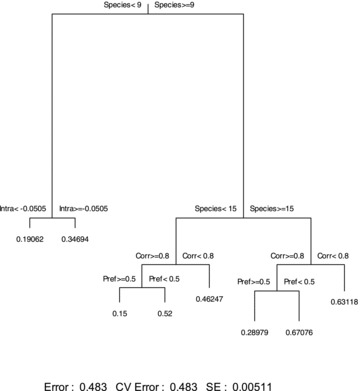										

For consumer species, the relationship between extinction risk and species richness differs between specialists (consumers that have strong preference for one of their resource species) and generalists (consumers that have equal preference for all their resource species). Overall, specialist consumers run a higher risk of extinction than do generalist consumers (Fig. 1a–d) (see also Tables A2 and A3 for regression tree analysis). Moreover, extinction risk for specialist consumers increases with increasing species richness while there is no clear trend for generalist consumers (Fig. 1a–d) (see also Tables A2 and A3 for regression tree analysis).

**Table A2:** Regression tree explaining the variation in extinction risk of herbivore species. Predictors: correlation among species in their response to environmental variation; prey preferences of consumers; species number; and strength of intraspecific competition in consumers. Overall, the tree explains 42.5% of the variation in the extinction risk.

Split								Variable and value(s)	Number of observations	Extinction risk
0								All data	24,000	0.419
	1							Preference ≥ 0.5 Generalists	12,000	0.303
		2						Intraspecific competition < –0.05	1,8000	0.188
				4				Correlation ≥ 0.6	1,3200	0.072
				4				Correlation < 0.6	1,4800	0.266
		2						Intraspecific competition ≥–0.05	1,4000	0.531
	1							Preference < 0.5 Specialists	12,000	0.535
			3					Species number < 15	1,6000	0.415
					5			Intraspecific competition < –0.55	1,2800	0.295
					5			Intraspecific competition ≥–0.55	1,3200	0.520
							7	Correlation < 0.25	1,1600	0.314
							7	Correlation ≥ 0.25	1,1600	0.726
			3					Species number ≥ 15	1,6000	0.655
						6		Correlation < 0.6	1,4400	0.593
						6		Correlation ≥ 0.6	1,1600	0.828
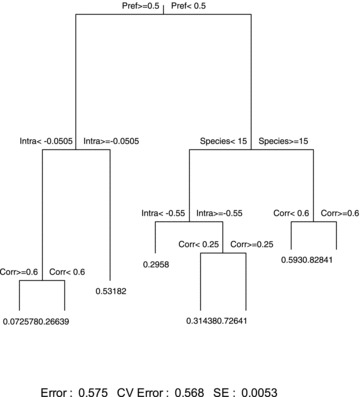										

**Table A3:** Regression tree explaining the variation in extinction risk of carnivore species. Predictors: correlation among species in their response to environmental variation; prey preferences of consumers; species number; and strength of intraspecific competition in consumers. Overall, the tree explains 24% of the variation in the extinction risk.

Split								Variable and value(s)	Number of observations	Extinction risk
0								All data	24,000	0.206
	1							Preference ≥ 0.5 Generalists	12,000	0.109
		2						Intraspecific competition < –0.05	1,8000	0.066
		2						Intraspecific competition ≥–0.05	1,4000	0.195
	1							Preference < 0.5 Specialists	12,000	0.303
			3					Species number < 9	1,3000	0.180
			3					Species number ≥ 9	1,9000	0.344
				4				Correlation < 0.25	1,4200	0.264
					5			Intraspecific competition ≥–0.55	1,2400	0.180
					5			Intraspecific competition < –0.55	1,1800	0.375
				4				Correlation ≥ 0.25	1,4800	0.414
						6		Intraspecific competition < –0.55	1,2400	0.265
							7	Correlation < 0.6	1,1200	0.123
							7	Correlation < 0.6	1,1200	0.406
						6		Intraspecific competition ≥–0.55	1,2400	0.563
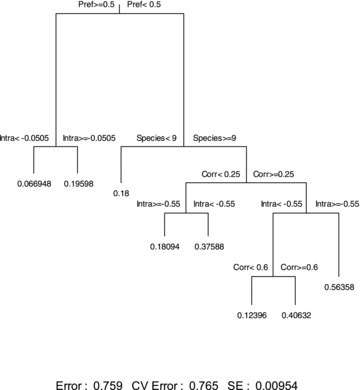										

**Figure 4:**
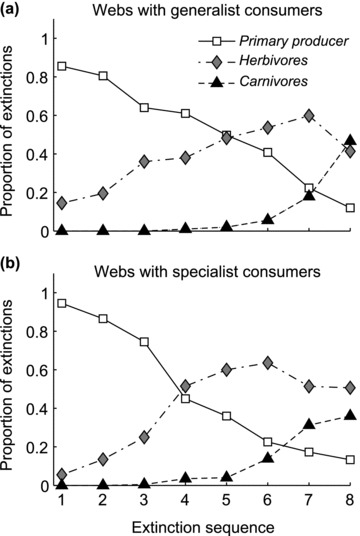
Proportion of extinctions affecting species at different trophic levels in the ordered sequence of extinctions in food webs with (a) generalist consumers and (b) specialist consumers. Extinctions early in the sequence are predominantly of primary producers, while extinctions late in the sequence are mainly of consumer species (herbivores and carnivores). Original number of species in the webs is equal to 12. Scenario: low correlation in species responses to environmental variation (ρ= 0.1), high environmental variation (var(ɛ) = 0.33), and weak intraspecific competition in consumers (*a_ii_=*–0.001). Results based on 200 independent replicate model food webs.

**Figure 5:**
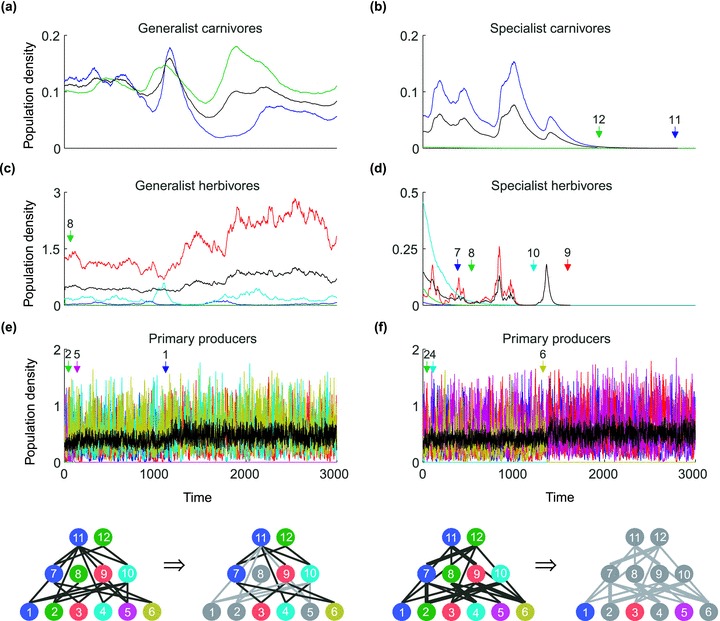
Examples showing the disassembly of 12-species food webs with generalist consumers (left panels) and specialist consumers (right panels). Top panels (a, b) show time series for carnivores, middle panels (c, d) show time series for herbivores, and bottom panels (e, f) show time series for primary producers. Black trajectories show time series for the mean density of species at each trophic level. Density compensation as well as compensatory dynamics can be seen in the primary producers. Time of species extinctions is indicated by arrows. Below the time series the specific food webs are displayed, before and after extinctions (extinct species are denoted by gray nodes and lost links by gray edges). Scenario: low correlation in species responses to environmental variation (ρ= 0.1), high environmental variation (var(ɛ) = 0.33), and weak intraspecific competition in consumers (*a_ii_=*–0.001).

Looking at the temporal pattern of extinctions—the order in which species from different trophic levels goes extinct—we find that primary producers are the first to go extinct, followed by herbivores, which in turn are followed by carnivores (Figs. 4 and 5). In other words, initial extinction of primary producers unleashes a bottom-up extinction cascade (see Fig. 5 for an example). Furthermore, the risk of such bottom-up extinction cascades—and hence extinction risk of both primary producers and consumers—is higher when correlation in the responses of species to environmental fluctuations is low than when correlation is high (Fig. 1; see also Tables A2 and A3 for regression tree analysis). To summarize, the risk of extinction cascades increases with increasing species richness and decreasing correlation among species in their responses to environmental variation, especially in food webs with specialist consumers.

**Figure 6:**
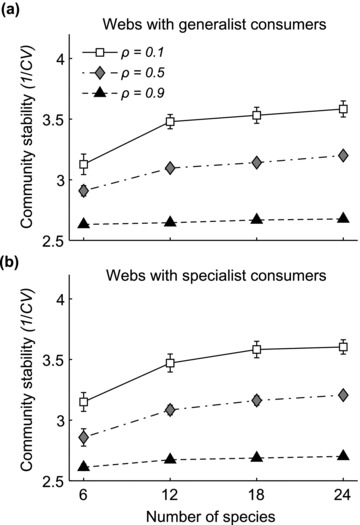
Temporal stability (1/CV) (bars show 95% CI) of aggregate abundance of primary producers in food webs with (a) generalist consumers and (b) specialist consumers. Series display the degree of correlation in species responses to environmental variation, ρ; ρ= 0.1 (solid line), ρ= 0.5 (dash-dotted line), and ρ= 0.9 (dashed line). Scenario: high environmental variation (var(ɛ) = 0.33) and weak intraspecific competition in consumers (*a_ii_=*–0.001). Results based on time series from 100 independent replicate model food webs.

**Figure A2:**
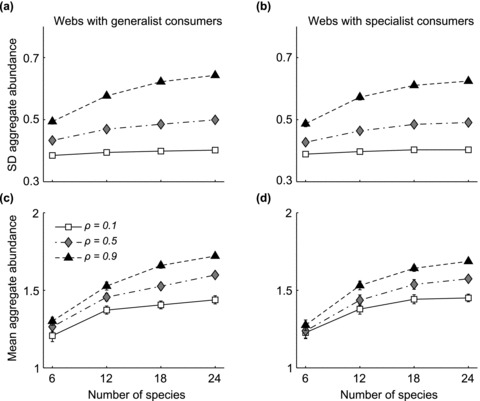
Standard deviation (a, b) and mean (c, d) of the aggregate abundance of primary producers as a function of species richness in webs with generalist (a, c) and specialist consumers (b, d). Bars show 95% CI. Series display the degree of correlation in species responses to environmental variation, ρ; ρ= 0.1 (solid line), ρ= 0.5 (dash-dotted line), and ρ= 0.9 (dashed line). Scenario: high environmental variation (var(ɛ) = 0.33) and weak intraspecific competition among consumers (*a_ii_=*–0.001). Results based on 100 independent replicate model food webs.

Mean density of primary producers varies less over time than the densities of individual primary producers (see Fig. 5 for an example), indicating compensatory dynamics of species. Furthermore, following extinction of primary producers, the mean density of the remaining primary producers increases (Fig. 5e and f), demonstrating the presence of density compensation (an inverse relationship between population density and species richness). Thus, the data from our analyses demonstrate the presence of compensatory dynamics among competing primary producers as well as density compensation following the extinction of species. Finally, we find that the temporal stability (measured as the reciprocal of the coefficient of variation, 1/CV; i.e., the mean divided by the standard deviation) of the aggregate abundance of primary producers increases with increasing species richness, especially when the correlation among species in their responses to environmental variation is low (Fig. 6). The main mechanism behind the increased temporal stability in our model food webs is overyielding, that is, an increased mean over time of the total abundance of all primary producers with increasing species richness (see Fig. A2). The standard deviation of combined abundances is nearly independent of species richness when correlation among species is low while it increases with species richness when correlation is high (see Fig. A2). These results are in line with the findings of a recent meta-analysis showing that the observed positive relationship between diversity and community stability in real communities is mainly associated with the overyielding effect (Jiang and Pu 2009).

## Discussion

Theoretical work suggests that extinction cascades and community collapses should be less likely to occur in species-rich multitrophic communities compared to species-poor ones in a constant and deterministic world, that is, in the absence of demographic and environmental variation (Ebenman et al. 2004; Dunne and Williams 2009). On the other hand, one study indicates that species-rich communities might be more sensitive to demographic stochasticity than species-poor ones (Ebenman et al. 2004). In small webs, the risk of quasi-collapse (the risk that the number of species will fall below a given level) was almost the same with and without demographic stochasticity, while in large webs risk of quasi-collapse was much higher with than without demographic stochasticity (Ebenman et al. 2004). However, the potential role of species richness for the robustness of food webs in highly variable environments is largely unknown.

Here, we find that the risk of cascading extinctions is higher in species-rich food webs than in species-poor ones in a highly variable environment. The most likely explanation for this is that the mean population density of primary producers decreases with increasing species richness, mainly because of increased intensity of interspecific competition (Fig. 2c). Thus, there is an inverse relationship between species richness and population densities—that is, density compensation (Lawler 1993; Ebenman et al. 2004; Borrvall and Ebenman 2008). As a consequence, primary producers will be closer to the extinction threshold in species-rich than in species-poor food webs resulting in increased risk of extinction. Here, we assume that each primary producer species competes with all other primary producer species. However, if each species only competes with a few “neighboring” species (i.e., niche-based competition), then the relationship between species richness and average densities of species might be much weaker (see Hughes and Roughgarden 2000). The density of a species will also be affected by predation pressure. When intraspecific competition in consumer species is weak, densities of consumers will be high leading to an increased predation pressure on primary producers (see Fig. 3). Thus, for a given species richness, primary producers will be closer to the extinction threshold when the strength of intraspecific competition in consumer species is weak than when it is strong (see Fig. 3).

Extinction risk of primary producers also increases with decreasing degree of correlation in the response of species to environmental fluctuations. Low correlation among primary producer species in their response to environmental fluctuations leads to low synchrony in their per capita growth rates (Table A4). Under these conditions, interspecific competition amplifies the environmentally driven population fluctuations leading to increased population variability of primary producers (May 1973b; Tilman 1996; Thebault and Loreau 2005). In the presence of high environmental variation, the amplitude of these fluctuations may become so large that the populations fall below the extinction threshold (see Fig. 5 for an example). Recent theoretical studies of competition communities (one trophic level) by Ruokolainen and colleagues suggest that our results are also valid for environments showing red noise (temporal autocorrelation). They found that high correlation among species in their response to environmental fluctuations decreased the probability of extinction both in the case of white and red noise (Ruokolainen et al. 2007; Ruokolainen and Fowler 2008; see Ruokolainen et al. 2009 for a review). The degree of correlation in the response among species to environmental fluctuations might depend on species richness. Specifically, response diversity (Elmqvist et al. 2003) is likely to increase with increasing species richness and hence correlation among species could be expected to decrease with increasing species richness. Our study suggests that this might lead to even higher extinction risks in species-rich communities, since low correlation per se leads to high extinction risks.

**Table A4:** Synchrony in per capita growth rates of primary producer species*.

Scenario (in-data)			Synchrony of basal species growth rate		
Prey preference	Correlation	Species richness	Average synchrony	Standard deviation synchrony	*n*
Skew	0.1	6	−0.04	0.05	97
Skew	0.1	12	0.01	0.06	100
Skew	0.1	18	0.01	0.07	100
Skew	0.1	24	0.03	0.06	100

Skew	0.5	6	0.38	0.04	98
Skew	0.5	12	0.38	0.05	100
Skew	0.5	18	0.38	0.05	100
Skew	0.5	24	0.37	0.07	100

Skew	0.9	6	0.85	0.02	100
Skew	0.9	12	0.84	0.02	100
Skew	0.9	18	0.84	0.02	100
Skew	0.9	24	0.84	0.02	100
Even	0.1	6	−0.03	0.06	98
Even	0.1	12	0.01	0.05	100
Even	0.1	18	0.01	0.07	100
Even	0.1	24	0.03	0.07	100

Even	0.5	6	0.37	0.04	97
Even	0.5	12	0.37	0.04	100
Even	0.5	18	0.38	0.05	100
Even	0.5	24	0.38	0.05	100

Even	0.9	6	0.85	0.01	99
Even	0.9	12	0.85	0.01	100
Even	0.9	18	0.85	0.01	100
Even	0.9	24	0.85	0.01	100

* Synchrony is the pairwise correlation (Pearson's linear correlation coefficient) over time between the per capita growth rates of two populations of primary producer species. For each replicate, one pair was chosen at random among the primary producer species in the web. The only criterion was that the species had to have survived for at least 100 time steps. This was to ensure that the time series would be long enough to give a reliable correlation value for the time series. In some cases, there were less than two species that fulfilled this criterion. Thus, synchrony could not always be calculated, leading to a sample size, *n*, of less than 100 (the number of replicates simulated). Average synchrony is the average correlation over all the replicates and standard deviation synchrony is the standard deviation of the same dataset.

An analysis of the temporal pattern of extinctions—the order in which species from different trophic levels goes extinct—reveals the mechanisms involved in the disassembly of the food webs. We find that primary producers are the first to go extinct, followed by herbivores, which in turn are followed by carnivores. Thus, the initial extinction of primary producers unleashes a bottom-up extinction cascade. Here, it is worth pointing out that the extinction risk of herbivores and carnivores increase with decreasing strength of intra-specific competition (see Fig. A1; see also Tables A2 and A3 for regression tree analysis). Weak intraspecific competition leads to high population densities of consumers (see Fig. 3). High densities should make consumers less, not more, vulnerable to stochastic processes, strongly suggesting that their extinctions are of a secondary, deterministic nature and due to the loss of primary producers. High densities of consumer species lead to high predation pressure on primary producers making primary producers more vulnerable to extinction, which in turn leads to increased risk of bottom-up extinction cascades.

The secondary nature of herbivore and carnivore extinctions is also demonstrated by the higher extinction risks of specialists compared to generalists. Specialist consumers are heavily dependent on one resource species as a source for nutrients and energy. As a consequence, the loss of the preferred resource species almost inevitably leads to extinction of the consumer species. Generalist consumers, on the other hand, are not similarly dependent on one particular resource species. A generalist consumer is therefore less likely to go secondarily extinct following the loss of one of its resource species. This is in line with the argument put forward by MacArthur already in 1955: consumer species feeding on many resource species should be less affected by variation in resource abundances than consumers feeding on few resource species. Now, since extinction risk of primary producer species increases with increasing species richness, we expect the extinction risk of specialist consumers to increase with increasing species richness as well. Extinction risk of generalist consumers, on the other hand, is not expected to be strongly related to species richness. This prediction is consistent with the results from our analysis and is further supported by a recent field experiment where it was found that population stability of specialist herbivores decreased with increasing plant species diversity while stability of generalist herbivores was unaffected or increased with increasing plant diversity (Haddad et al. 2011).

We find that the temporal stability of the aggregate abundance of primary producers increases with increasing species richness, especially when the correlation among species in their responses to environmental variation is low. This is in line with the insurance hypothesis (Yachi and Loreau 1999; Elmqvist et al. 2003), which states that community level stability should increase with increasing species richness if species respond differently (low correlation) to environmental fluctuations. Thus, in this respect our results corroborate earlier theoretical studies suggesting that the insurance hypothesis should also be effective in multitrophic communities (Ives et al. 2000; Thebault and Loreau 2005). A recent meta-analysis of empirical data further supports this prediction (Jiang and Pu 2009). Although we find the temporal stability of total primary producer abundance to increase with species richness, we also find the risk of cascading extinction to increase. Thus, the effects of biodiversity on the response of ecosystems to an increasingly variable environment is two-sided: exactly the same conditions—high species richness and low correlation in the responses of species to environmental variation—that lead to increased temporal stability of aggregate producer abundance result in increased risks of extinction cascades. How can high temporal stability at the community level be reconciled with a high extinction risk at the species level? We find that even though extinction risk per species is higher in species-rich communities than in species-poor ones, they still have more species remaining in the postextinction communities than do the species-poor communities. As a result, they will still have a higher compensatory capacity than the species-poor communities. However, in the long term, extinction cascades may undermine the compensatory capacity of ecosystems and hence lead to decreased stability of ecosystem processes such as primary production.

To conclude, our theoretical study suggests that global warming and accompanying increased levels of environmental variability should have a more negative impact on the coexistence of interacting species in species-rich communities than in species-poor ones, especially when species respond independently to environmental variation. In a way, our results contrast with earlier work suggesting that environmental variation might promote coexistence of competing species if species differ in their response to this variation (e.g. Chesson and Warner 1981). A likely reason for the contrasting predictions is that we here account for processes such as demographic stochasticity and Allee effects by defining quasi-extinction thresholds. These processes cause a disadvantage to rare species and hence make coexistence less likely (Adler and Drake 2008; Gravel et al. 2011). Since population densities decrease with increasing species richness, species in species-rich communities should be relatively more exposed to these processes and hence more likely to go extinct (MacArthur 1972). These arguments are supported by an experimental study of microcosm communities (bacteria and protists) where it was found that species-rich communities had lower population densities and more extinctions than species-poor communities (Lawler 1993).

We would like to end by noting that the average number of links per species (link density) increases with increasing species richness in our model food webs since we keep connectance constant. Thus, on average, a consumer species in a species-rich web has more prey species than a consumer in a species-poor web. Should we instead keep link density constant (i.e., connectance decreases with increasing species richness) that would mean that consumer species in species-rich webs will, on average, have the same number of prey species as consumers in species-poor webs. Preliminary analysis indicates that constant link density would lead to an even higher risk of extinction cascades in species-rich food webs compared to species-poor ones. The relationship between link density and species richness in real food webs is largely unknown. One recent study found a positive relationship between the proportion of extreme specialists (species using only one resource type) and species richness in three ecological communities (Vázquez and Stevens 2004). In light of our results, this implies that highly species-rich ecosystems, such as tropical rainforests and coral reefs, might be particularly vulnerable to increased levels of environmental variability and hence to increased intensity and frequency of weather extremes caused by global warming.

## Appendix

### Food web model

Food web dynamics are described by a generalized Rosenzweig–MacArthur model with stochastic parameters (Rosenzweig and MacArthur 1963; Borrvall and Ebenman 2008):

where *dN_i_*/*dt* is the rate of change of density of species *i* with respect to time in a community with *s* species, *b_i_*(*t*) is the intrinsic per capita growth (mortality) rate for primary producer (consumer) species *i* at time *t*, and  is the per capita effect of species *j* on the per capita growth rate of species *i*. The functional response is of type II:

where *h_ij_* is the preference of predator *j* for prey *i*,  is the intrinsic attack rate of predator *j* on prey *i*, *T* is the handling time needed for the predator to catch and consume the prey, and *e* is a measure of conversion efficiency, that is, the rate at which resources are converted into new consumers. *L*(*i*) is the set of species belonging to the same trophic level as species *i*, *C*(*i*) contains the species that consume species *i*, and *R*(*i*) contains the species being resources to species *i*. Intraspecific competition is given by *a_ii_* and interspecific competition among primary producers is given by *a_ij_*.

Environmental stochasticity is introduced as white noise (i.e., no serial correlation) affecting the intrinsic growth rates (mortality rates); . Here  is the mean value of the intrinsic growth rate (mortality rate) of species *i* and *ɛ_i_*(*t*) is a stochastic process. The stochastic processes are approximated as piecewise constant functions in time *ɛ_i_*(*t*) =*ɛ_ik_*, *k**t* < *k*+ 1 (*k*= 0, 1, …), that is, with a constant value during each time unit, *k*. The values for *ɛ_ik_* are drawn from a uniform distribution with minimum, mean, and maximum values equal to –1, 0, and 1, respectively. The correlation, ρ, among time series of *ɛ_ik_* are varied from ρ= 0.1 to 0.9 in steps of 0.2 to model different response to environmental fluctuations among species. We generate correlated multidimensional Gaussian copulas that are transformed with the normal cumulative distribution function and rescaled to obtain the desired time series. The Gaussian copulas are obtained using a Cholesky factorization of the covariance matrix as implemented in the Matlab function mvnrnd. Spearman's rank correlation is used to adjust the correlations for the Gaussian copulas in order to obtain the desired correlation between the uniform distributions for species responses. The resulting coupled ordinary differential equations (ODEs) are integrated in time using a variable order solver based on numerical differentiation formulas (NDFs), as implemented in the Matlab function ode15s. The solver is restarted at each time unit to avoid numerical problems due to the steps in the stochastic piecewise constant growth (mortality) rates.

### Parameterization

Model parameters were chosen as follows. For primary producers, mean intrinsic growth rate, , is set to 1. For consumers, mean intrinsic mortality rate, , is randomly drawn from the uniform distribution [–0.01, 0]. Mortality rates for consumers are sorted, resulting in lower rates for carnivores compared with their prey, that is, . This is because body size often increases with trophic level (Jonsson et al. 2005), and larger body sizes often confer lower mortality rates (Roff 1992). The strength of intraspecific competition is set equal to –1 for basal species. For consumer species, we have explored two scenarios: (1) strong intraspecific competition, *a_ii_*=–1, and (2) weak intraspecific competition, *a_ii_*=–0.001. The strength of interspecific competition among basal species is randomly drawn from the uniform interval [–0.7, –0.3]. Each consumer has a maximum prey preference equal to 1 (i.e., consumers with only one prey has *h_ij_*= 1). For consumers with more than one prey, we investigate two scenarios: (1) consumers are assumed to have a high preference (0.9) for one of their prey species (assigned randomly) and equal lower preferences (0.1 shared equally among the rest of the prey species) for the others; (2) consumers have equal preferences for all their prey species (1 shared equally among all prey species). In case (1), consumer species can be considered as specialists and in case (2), as generalists. For each consumer–prey interaction, the intrinsic attack rate, *α_ij_*, is randomly drawn from the uniform distribution [0, 1]. Handling time, *T*, is assumed to be the same for all consumers on all prey and is given the value of 0.01. Conversion efficiency, *e*, is set to 0.2 for links between adjacent trophic levels (*e* < 1 when the size of the consumer is larger than that of its prey). Omnivory links are assumed to be less efficient and we therefore use *e*= 0.02 for these links.

### Persistence and quasi-extinction thresholds

To attain some generality, we generate a large number of replicate communities with constrained randomization of links and parameters (see also Borrvall and Ebenman 2006, 2008). We keep generating replicates until 200 replicate communities that are persistent in a deterministic environment have been found. Communities are considered persistent if all species densities remain above predefined thresholds (see below) following numerical integration over 50,000 time units using a deterministic model with the per capita growth rates set to the mean values. By using this procedure, we check that communities would persist in a deterministic setting (constant environment). Then, environmental stochasticity is added to each of the 200 persistent deterministic replicate communities and the systems are simulated for 10,000 time units. During the simulation, all species are checked for extinction. A species is considered extinct if its density falls below a specified quasi-extinction threshold. Top consumers (species without predators) that lose all their prey species are deleted from the web immediately. (They no longer interact with any other species.) Intermediate species (herbivores being consumed by carnivores) that lose all their resources are deleted after 50 time steps.

The quasi-extinction thresholds are, on average, approximately two orders of magnitude smaller than the equilibrium densities of the respective species categories (primary producers, herbivores, and carnivores) in webs with strong intraspecific competition in consumers. In webs where intraspecific competition in consumer species is weak, their equilibrium densities are higher and hence further away from the extinction threshold. The exact sizes of the extinction thresholds are not of primary importance here since we are mainly interested in investigating relative extinction risks for different scenarios (species-rich vs. species-poor communities; low vs. high correlation in species responses to environmental variation; generalist vs. specialist consumers; weak vs. strong intraspecific competition in consumer species) rather than in predicting absolute extinction risks.

### Synchrony in per capita growth rates of primary producers

We measure synchrony using the pairwise correlation (Pearson's correlation coefficient) over time between the per capita growth rates of two populations of primary producer species. For each replicate, one pair was chosen at random among the primary producer species in the web. The only criterion was that the primary producer species had to have survived for at least 100 time steps. This was to ensure that the time series would be long enough to give a reliable correlation value for the time series. Average synchrony is the average correlation over all the replicates fulfilling the mentioned criterion (Table A4).
